# strainedSMILES2xyz: a workflow for reliable 3D structures of strained molecules from SMILES

**DOI:** 10.1186/s13321-026-01219-0

**Published:** 2026-05-16

**Authors:** Tori Demuth, Julian Schnizer, Dennis Svatunek

**Affiliations:** Institute of Applied Synthetic Chemistry, TU Wien, Getreidemarkt 9, 1060 Vienna, Austria

**Keywords:** Ring strain, Conformer generation, RDKit, Bioorthogonal chemistry, Cycloalkynes, trans-Cycloalkenes, SMILES, XYZ

## Abstract

Accurate 3D structure generation from SMILES is essential for data-driven chemistry but often fails for strained ring systems. We introduce **strainedSMILES2xyz**, a Python workflow that improves conformer generation by relaxing RDKit constraints, exploring stereoisomer variants, and correcting errors using force-field refinement. Benchmarking on strained and unstrained rings shows that it outperforms existing tools, generating correct geometries in nearly all cases. The workflow is available as a Python package and Jupyter notebook.

**Scientific Contribution**

This work identifies a critical gap in automated SMILES-to-3D structure generation for strained molecules. Many established tools, including the widely used RDKit, frequently fail or produce incorrect geometries for these systems. By explicitly targeting these failure modes, the proposed approach enables reliable 3D structure generation for chemically relevant strained molecules within fully automated workflows.

## Introduction

The automated generation of 3D molecular geometries from SMILES strings has become a cornerstone of modern computational chemistry. With the rise of large-scale virtual screenings, machine learning models, and data-driven chemical design, the need for reliable, fully automated structure generation pipelines is more critical than ever. Tools such as RDKit [1] provide robust conformer generation for many classes of molecules and are often used as backends for further quantum chemical workflows, including those for transition state searches, like the autodE [2] and similar packages.

**Fig. 1:**
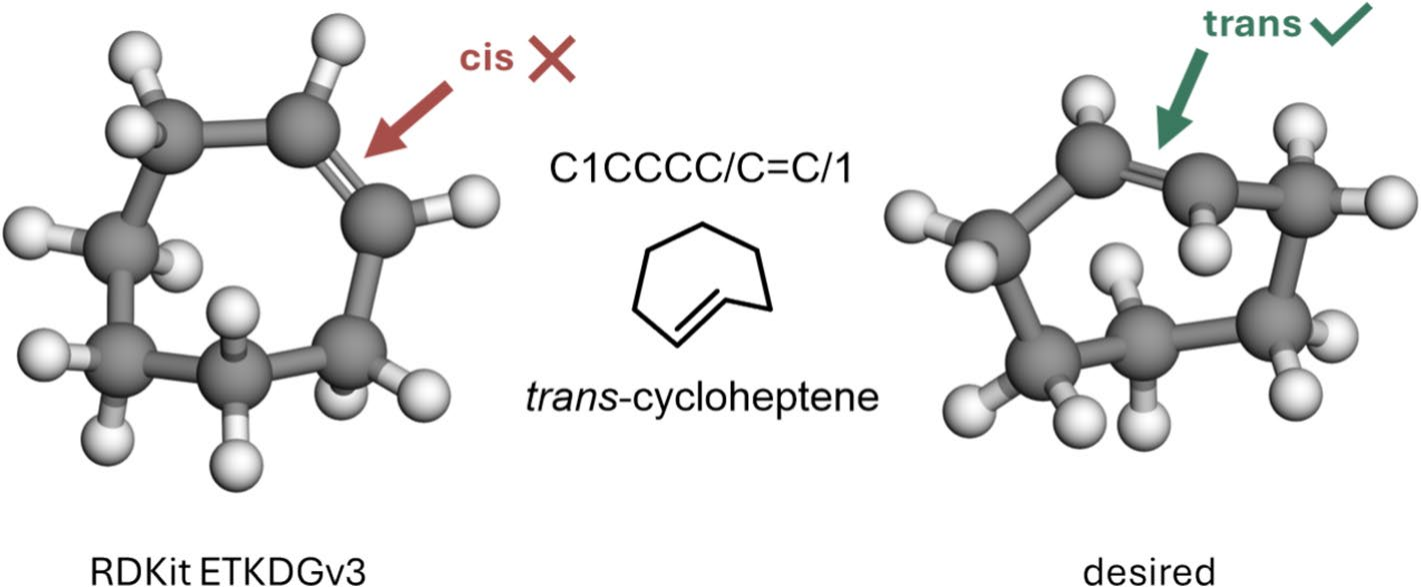
RDKit produced and desired geometry for SMILES C1CCCC/C = C/1 (*trans*-cycloheptene)

However, we observed a consistent and critical failure point in these tools: strained ring systems. These comprise small rings, bridged systems, and geometrically constrained scaffolds that frequently appear in modern organic chemistry. This includes bioorthogonal chemistry, where these scaffolds play key roles as both transient intermediates and stable compounds. This class of molecules has recently seen increasing attention in computational studies by us and others [3–20], underscoring the need for reliable computational treatment. Yet, we found that pipelines based on RDKit, Open Babel, and related tools often fail, either by generating the wrong geometries or by failing to produce any structure at all. An example of a wrong geometry is shown in Fig. 1. Here RDKit produces the *cis*-cycloheptene instead of the *trans*-cycloheptene. For *trans*-cyclooctene it failed completely and produced no geometry at all.

Given the increasing importance of these strained systems in both discovery and mechanistic studies, and our own ongoing work in this area, we developed a dedicated workflow, **strainedSMILES2xyz**, that builds on RDKit for initial handling but augments it with quantum chemical structure refinement using ORCA [21, 22]. This ensures that even highly strained systems can be treated in a fully automated manner, yielding correct 3D geometries suitable for downstream applications. In this paper, we introduce this workflow and demonstrate its utility and robustness across a range of strained ring systems.

### Implementation

**Fig. 2:**
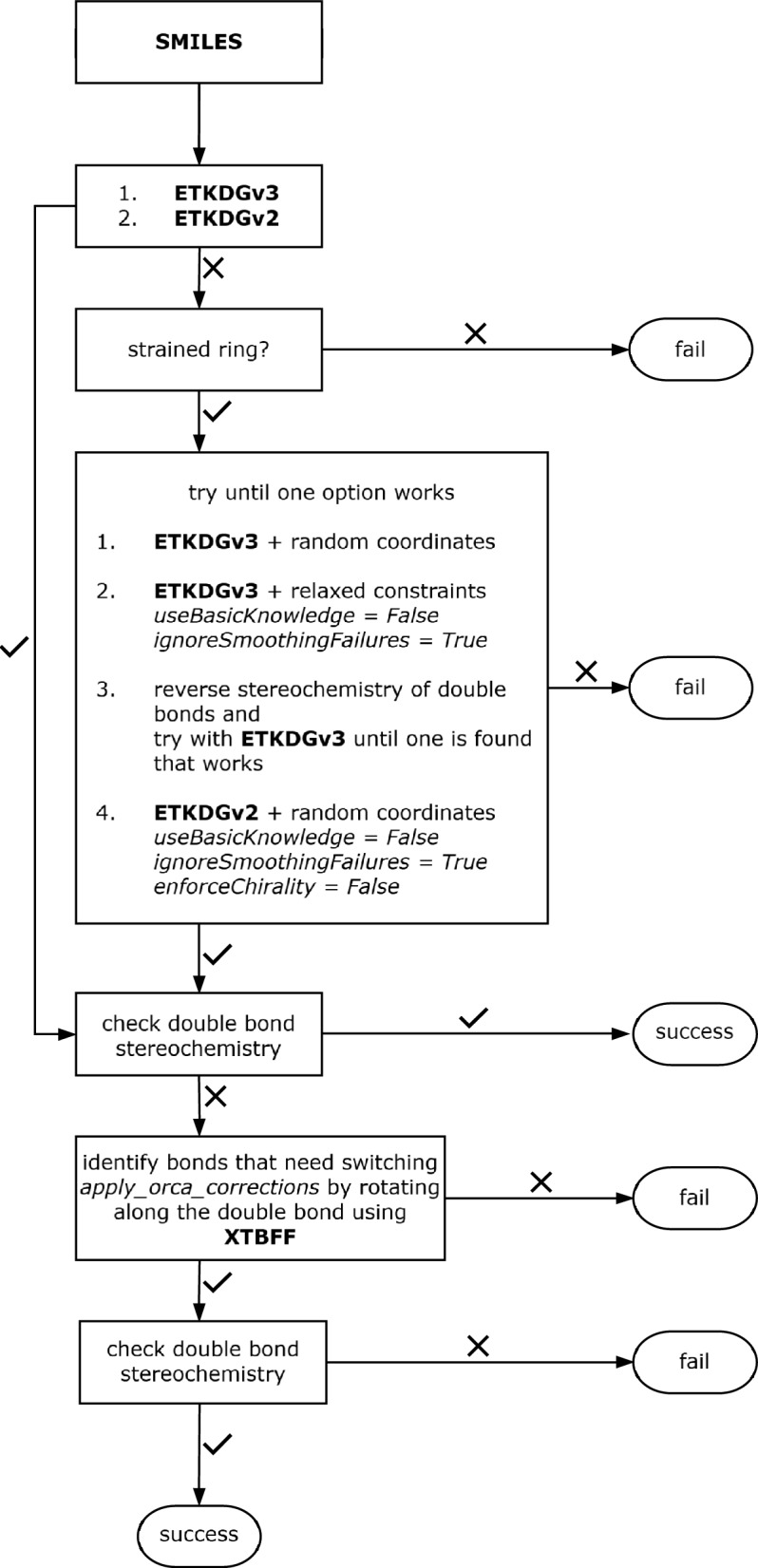
Flowchart of the **strainedSMILES2xyz** workflow

Figure 2 illustrates a high-level overview of the **strainedSMILES2xyz** workflow. The process begins with the conversion of a SMILES string into a 3D structure using RDKit. Conformer generation is first attempted using the ETKDGv3 and ETKDGv2 algorithms [23]. If both fail, the molecule is analyzed for the presence of strained ring systems, which we define as rings containing fewer than twelve atoms and at least one double or triple bond.

In the presence of such strain, the workflow proceeds through a series of increasingly aggressive strategies.

First, we attempt ETKDGv3 again, this time using random initial coordinates. If that fails, we relax the conformer generation constraints further by disabling the use of basic chemical knowledge and instructing RDKit to ignore smoothing failures. Here, a smoothing failure refers to a situation where the interatomic distance constraints cannot be made mutually consistent, such that no valid 3D embedding satisfying all constraints exists. As a next step, if the molecule contains one or more double bonds with defined stereochemistry, we systematically invert each double bond, generating all possible cis/trans combinations consistent with the molecular graph, and retry ETKDGv3 for each variant until we are able to generate a structure. This approach accounts for cases where the stereochemistry specified in the SMILES is physically reasonable but cannot be achieved by RDKit’s ETKDGv3 algorithm due to its internal constraints. By systematically inverting double bond configurations and retrying the embedding, we increase the chances of generating a wrong stereoisomer that can subsequently be corrected to the intended structure.

Should none of these strategies succeed, the workflow resorts to a final attempt where random coordinates are enabled, chirality enforcement is disabled, the use of basic knowledge is again suppressed, and smoothing failures are ignored. This stage is explicitly flagged as it may introduce errors at stereocenters unrelated to the double bonds of interest.

If any of these attempts yield a 3D geometry, the resulting structure is compared against the input SMILES to verify double bond stereochemistry. If all stereochemical assignments are consistent, the structure is accepted. Otherwise, a correction routine is invoked. In this routine, incorrectly assigned double bonds are identified and subjected to relaxed scan rotation using ORCA’s GFN-FF force field (also referred to as XTBFF). In a final step, the stereochemistry is then re-evaluated and either accepted or rejected.

The workflow is available both as a Python package and as a Jupyter notebook, allowing for interactive testing or integration into automated pipelines. The main entry point accepts a SMILES string via the smiles argument and attempts to generate a 3D structure with correct stereochemistry. The level of logging output can be adjusted using print_level, and the behavior of RDKit's conformer generation can be modified by setting no_chirality_enforced_allowed to disallow the fallback strategy with no enforced chirality. If use_orca_corrections is set to True, the workflow will perform stereochemistry corrections using ORCA with the GFN-FF force field [24] when mismatches are detected. The environment variable ORCA_PATH must be set to the path of the ORCA executable to enable geometry correction via ORCA. The number of CPU cores used for ORCA calculations can be specified via n_cores. The output includes a 3D RDKit mol object and a dictionary of metadata summarizing whether corrections were necessary and which strategies were applied.

All computations were performed using Python 3.11.23, with the following key packages: RDKit 2025.03.4, NumPy 2.3.1, Pandas 2.3.0, py3Dmol 2.5.1, and Open Babel 3.1.1 (Python interface). Geometry optimizations and stereochemical corrections were carried out with ORCA 6.1.0 [22]. For testing, the workflow was performed on systems using Ubuntu 24.04.

## Results

**Fig. 3:**
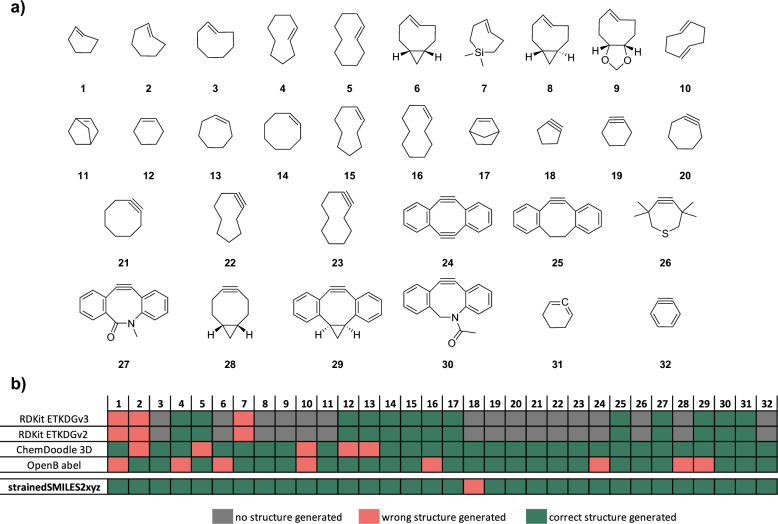
**a** 2D structures of the benchmark data set. **b** Comparison of structure generation results across different methods.

To evaluate our approach, we constructed a benchmark dataset consisting of 32 strained and unstrained ring systems. Figure 3a displays the 2D structures of all test molecules. Many of these compounds were taken from literature and represent chemically relevant or experimentally studied molecules, ensuring that the benchmark reflects real-world cases encountered in synthetic and bioorthogonal chemistry. The dataset includes *trans*-cycloalkenes, cycloalkynes, and other small, strained ring systems. For example, compound **11** is a recently reported anti-Bredt alkene [3], and compound **7** corresponds to the sila-*trans*-cycloheptene developed by Joseph Fox [25].

Representative scaffolds from bioorthogonal chemistry are also included: compound **3** is the core of *trans*-cyclooctenes (TCOs), **6** represents more reactive strained *trans*-cyclooctene (sTCO), and **9** corresponds to dioxolane-fused *trans*-cyclooctene (dTCO). Additionally, common cyclooctynes such as DBCO (**25**), BCN (**28**), ADIBO (**30**), and the recently introduced DMBO (**29**)[4, 26] are part of the set.

We then applied a range of common structure generation tools, RDKit (ETKDGv3 and ETKDGv2), Open Babel, ChemDoodle 3D, and our **strainedSMILES2xyz** workflow, to generate 3D geometries from SMILES strings. All resulting structures were subsequently optimized using the GFN2-xTB[27] method in ORCA to ensure fair and realistic comparison, and then manually evaluated for correctness.

Figure 3b summarizes the benchmark results. Grey indicates cases where structure generation failed entirely, red highlights structures that were generated but had incorrect connectivity or stereochemistry (the most critical failure), and green indicates correctly generated structures. RDKit (both ETKDGv3 and ETKDGv2) failed to generate valid geometries for a substantial number of cases and produced incorrect structures in three instances. While Open Babel and ChemDoodle 3D succeeded in generating geometries for all molecules, they also produced a higher number of incorrect geometries. In contrast, RDKit more frequently failed to generate a structure but produced fewer incorrect results among successful cases. From a workflow perspective, failed structure generation is preferable to incorrect geometries, as failures can be detected and handled explicitly, whereas incorrect structures may propagate undetected into downstream calculations.

In contrast, strainedSMILES2xyz successfully generated geometries for all test cases and produced incorrect output in only a single case: cyclopentyne. This highly strained alkyne has been observed as a fleeting intermediate in synthetic chemistry but is known to be extremely unstable [28]. As such, we consider this a tolerable failure, given that molecules of this type are unlikely to be suitable for automated screening or conformer generation pipelines. Overall, this represents a substantial improvement in reliability over existing tools.

**Fig. 4:**
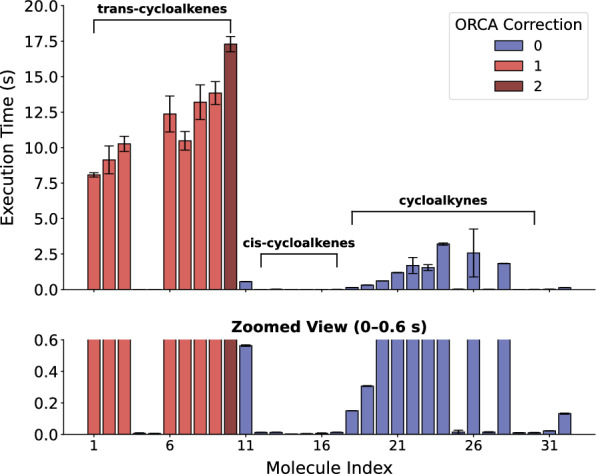
Execution times for 3D coordinate generation of the benchmark molecules using **strainedSMILES2xyz**, shown with a split y-axis (upper: full range, lower: zoomed-in view of shorter times). Bars are color-coded by the number of double-bond stereochemistry corrections applied using the ORCA-based procedure (0, 1, or 2 corrections). Molecules are grouped into *trans*-cycloalkenes, *cis*-cycloalkenes, and cycloalkynes

To investigate execution times of the new method, **strainedSMILES2xyz** was run in triplicate for each of the 32 benchmark molecules, and execution times were recorded (Fig. 4). Detailed results are provided in *timings.csv*. Benchmarks were performed using 16 cores on a Threadripper 3970X system.

For *trans*-cycloalkenes, stereochemical correction of the double bond was sometimes required, leading to total execution times between 7.5 and 20 s. If no correction was necessary, execution times were in the millisecond range. *cis*-Cycloalkenes could be generated directly with the default algorithm, requiring only a few milliseconds. In contrast, cycloalkynes sometimes require several seconds to generate, even without force-field corrections. A clear difference was observed between dibenzocycloalkynes, which were generated within milliseconds, and other cycloalkynes, which took considerably longer. This slowdown is due to RDKit algorithms needing more attempts and retries to generate geometries for highly strained systems, especially when executed multiple times under different settings as defined by our workflow.

Overall, execution times of up to 20 s are acceptable, demonstrating that our workflow performs as fast as RDKit for easily generated molecules and adds only moderate overhead for more challenging cases.

## Discussion

For strained alkynes, the primary failure mode of RDKit was the inability to generate any geometry at all, owing to the extreme strain in these systems. To address this, we relaxed several of RDKit’s ETKDGv3 safety features, specifically, by disabling useBasicKnowledge and allowing ignoreSmoothingFailures. While the resulting initial geometries were often highly distorted, a quick geometry optimization using GFN2-xTB was sufficient to recover realistic structures in all but one case. 

For cycloalkenes, disabling RDKit’s safety features improved the likelihood of generating a geometry, even if the stereochemistry was incorrect; however, in some cases, no structure could be generated at all. To address this, we implemented a strategy of explicitly generating possible double bond stereoisomers by inverting cis/trans configurations. This enabled successful geometry generation in all cases. However, due to the combination of relaxed safety constraints and deliberate stereochemical manipulation, the resulting geometries frequently displayed incorrect double bond configurations.

To correct these, we implemented a verification step comparing the generated 3D structure to the input SMILES and, if discrepancies were found, attempted to invert the stereochemistry by rotating around the double bond.

Initial attempts to rotate C = C double bonds directly using GFN2-xTB often failed. The main issue was that residues not defined by the dihedral angle did not rotate appropriately, leading to failed optimizations or even incorrect connectivities in the resulting structure. We identified the high rotational barrier of the C = C bond as the underlying cause. As a workaround, we experimented with temporary substitution of the double bond, for example, replacing the C = C with B–N or C⁺–N units that have some double bond character but are more flexible. This improved rotational behavior but introduced a new problem: increased likelihood of rearrangements or group migration, similar to carbocation rearrangement reactions, resulting in incorrect molecular connectivity.

To avoid these issues, we transitioned to a classical molecular mechanics force field, where topology is fixed by design, and found GFN-FF to prove effective. It provided sufficient flexibility to perform double bond rotations while preserving the molecular connectivity, making it well-suited for this type of correction.

## Conclusions

Reliable 3D structure generation from SMILES is a critical step in data-driven chemistry, yet existing tools frequently fail when applied to strained ring systems. We developed **strainedSMILES2xyz**, a workflow that systematically overcomes these limitations by applying progressively relaxed conformer generation strategies and, when needed, stereochemistry corrections using force-field-based refinement. Benchmarking against a curated dataset of strained and unstrained rings, many of which are chemically relevant structures drawn from the literature, demonstrated that **strainedSMILES2xyz **consistently outperforms established tools such as RDKit, Open Babel, and ChemDoodle 3D. The workflow was able to generate correct 3D geometries for all but one challenging system, and it represents a robust solution for reliably handling geometrically complex molecules in automated pipelines.

The method is made freely available as both a Python package and an interactive Jupyter notebook, supporting further adoption and extension in computational chemistry workflows.

## Availability and requirements

· **Project name:** strainedSMILES2xyz.

· **Project home page:** https://github.com/Svatunek-Lab/strainedSMILES2xyz, https://pypi.org/project/strainedsmiles2xyz/

· **Operating system(s):** Platform independent.

· **Programming language:** Python.

· **Other requirements:** RDkit ≥ 2025.03.4, ORCA ≥ 6.0.0

· **License:** GPL-3.0

**Any restrictions to use by non-academics:** ORCA is free of charge only for academic research and teaching purposes.

## Data Availability

All scripts, Jupyter notebooks, resulting XYZ files, and the final strainedSMILES2xyz source files are available at https://github.com/Svatunek-Lab/strainedSMILES2xyz and are archived on Zenodo.
